# Using the *capabilities approach* to understand inequality in primary health-care services for people with severe mental illness

**DOI:** 10.1186/s13584-018-0236-x

**Published:** 2018-08-27

**Authors:** Maya Lavie-Ajayi, Galia S. Moran, Itzhak Levav, Rotem Porat, Tal Reches, Margalit Goldfracht, Gilad Gal

**Affiliations:** 1 The Israeli Center for Qualitative Research of People and Societies, Ben-Gurion University of the Negev, P.O.B. 653, 8410501 Beer-Sheva, Israel; 2 The Spitzer Department of Social Work, Ben-Gurion University of the Negev, P.O.B. 653, 8410501 Beer-Sheva, Israel; 3 Department of Community Mental Health, University of Haifa, 199 Aba Khoushy Ave, Haifa, Israel; 4 Quality Improvement Department, Clalit Health Services, Tel Aviv, Israel; 5 School of Behavioral Sciences, Tel Aviv-Yaffo Academic College, Tel Aviv, Israel

**Keywords:** Severe mental illness, Capability approach, Health services, Primary health care

## Abstract

**Background:**

Epidemiological studies show disparities in the provision of physical health-care for people with severe mental illness. This observation includes countries with universal health insurance. However, there is limited in-depth data regarding the barriers preventing equality of physical health-care provision for this population. This study applied the capabilities approach to examine the interface between general practitioners and patients with severe mental illness. The capabilities approach provides a framework for health status which conceptualizes the internal and external factors relating to the available options (capabilities) and subsequent health outcomes (functioning).

**Methods:**

Semi-structured in-depth interviews were conducted with 10 general practitioners and 15 patients with severe mental illness, and then thematically analyzed.

Results: We identified factors manifesting across three levels: personal, relational-societal, and organizational. At the personal level, the utilization of physical health services was impaired by the exacerbation of psychiatric symptoms. At the relational level, both patients and physicians described the importance of a long-term and trusting relationship, and provided examples demonstrating the implications of relational ruptures. Finally, two structural-level impediments were described by the physicians: the absence of continuous monitoring of patients with severe mental illness, and the shortfall in psychosocial interventions.

**Conclusion:**

The capability approach facilitated the identification of barriers preventing equitable health-care provision for patients with severe mental illness. Based on our findings, we propose a number of practical suggestions to improve physical health-care for this population: **1**. A proactive approach in monitoring patients’ health status and utilization of services. **2**. Acknowledgment of people with severe mental illness as a vulnerable population at risk, that need increased time for physician-patient consultations. **3**. Training and support for general practitioners. **4**. Increase collaboration between general practitioners and mental-health professionals. **5**. Educational programs for health professionals to reduce prejudice against people with severe mental illness.

## Background

People with severe mental illness (SMI) are more likely to have poorer physical health and are at increased risk for premature death associated with comorbid somatic conditions such as diabetes and cardiovascular disease [1–6]. Despite this heightened risk, studies have also shown medical care disparities for these patients [5]. Inequalities in the physical health-care provision for people with SMI play a major role in what has been described as “the scandal of premature mortality” [7]. Health disparities have been documented in the United States for SMI populations protected by special insurance systems (e.g., army veterans) [8, 9], as well as in countries with national health insurance schemes, where care is not dependent upon out-of-pocket expenditure, e.g., Australia [4], Canada [10], Israel [11], Sweden [12] and Taiwan [13].

The transition from psychiatric hospitalization to community services places general practitioners (GPs) in a central position – and primary care as the first and ongoing point of contact – for many individuals with SMI. This makes the interaction between GPs and patients with SMI an important locus for the study of physical health-care provisions for this population [14]. This proposed research strategy makes it possible to compare the data derived from GPs and patients, highlight common and contradictory themes, and provide a richer and more holistic perspective with regard to both parties [15]. However, only limited in-depth studies, drawing from the perspectives of these two principal stakeholders, have been conducted with a view to understanding access to, and utilization of, primary care services by people with SMI [16]. One study found that both GPs and patients with SMI view longitudinal relationships as vital for high-quality care [17]. Another study reported on barriers associated with the patients (e.g. socioeconomic and psychological barriers), GPs (e.g. knowledge and personal values) and the health system (e.g. models of primary care delivery) [18].

In order to extend existing knowledge about barriers to primary care, this study applied the health capability approach (CA), providing a theoretical framework for the interpretation of the findings.

### The health capability approach

The core characteristic of the CA is its focus on what people are effectively able to do and to be, i.e. their capabilities (19). When applied to the health domain, CA highlights the capabilities, or genuine opportunities, that are available for people in order to be healthy. It focuses on the process of converting goods (e.g., health services, personal resources) into opportunities. Specifically, it assesses what people can actually achieve with the resources at their disposal while taking into account social diversity, abilities and circumstances. The CA model is unique in its ability to describe how structural conditions, materials, and normative constraints shape individuals’ opportunities to access and utilize healthcare [19] and offers an alternative framework to utilitarian (resource- or income-based) approaches to human welfare [19, 20].

**Fig. 1:**
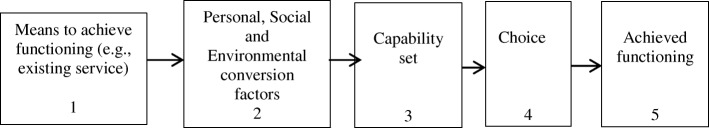
Schematic representation of the links between existing services, a person’s capability set, and achieved functioning. Based on capability approach figure one represents the links between Means to achieve functioning (e.g., existing service), Personal, Social and Environmental conversion factors, Capability set, Choice and Achieved functioning

The health CA further conceptualizes factors that can enhance or impede one’s capabilities and functioning in terms of a range of personally valued wellbeing and practical states [20, 21]. “Capability” refers to the options and genuine opportunities available to individuals to realize functioning. “Functioning” is the outcome of actions undertaken to maintain or improve one’s health. “Conversion factors” refer to the internal and external aspects affecting one’s opportunities [21]. Whilst all conversion factors influence how a person may be able to convert characteristics and resources into improved functioning, the sources of these factors may differ. They include: personal conversion factors, such as individual characteristics (e.g., physical, mental, cognitive), that can affect the potential to exploit available resources or services; social conversion factors, consisting of the norms or power relations that influence resource usage behavior; and environmental conversion factors, which relate to the infrastructure, laws, and barriers that are external to the individual agency. Each of these factors can affect the options/possibilities to utilize goods and services in order to achieve the desired functioning (Fig. 1).

To illustrate this theory with an example from the physical disability realm, we consider the service of the GP clinic as the *means* (Box 1 in Fig. 1) to the desired end, namely, health functioning. This person’s impairment, which limits him/her from physically attending his GP’s clinic, is labeled as a *personal conversion factor* (Box 2). A potential *social conversion factor* is made up of the authority of the GP – the person authorized to prescribe the medications needed by the patient – combined with the GP’s distant, formal approach to the physically disabled patient (Box 2). Finally, the lack of physical access for wheelchair users to the GP’s clinic may function as an *environmental conversion factor* (Box 2). Together, these conversion factors constitute barriers, preventing patients from accessing and utilizing the full range of available, high-quality GP services (Box 3); limit the extent of the patient’s choice (Box 4), and prevent him from achieving optimal health functioning (Box 5).

The example above of a quadriplegic person was an illustration of a negative track of the CA theory. We next consider a positive track of CA, with an example based on a different person - one who has a mental health disability. Notably, if this person is in remission, has a trustworthy relationship with the GP and has access to resources about legal rights, then the conversion factors will allow him to achieve and exercise functioning. Thus, the concept of “capability” refers to the feasible opportunities constrained by internal (personal) and external (social and environmental) conversion factors that can have either positive or negative implications on one’s health outcomes [21].

### Objectives

In common with other countries, health disparities experienced by people with SMI in Israel have been reported [11]. This study seeks to examine the GP-patient interface by employing a dual perspective guided by the CA framework, in order to understand the barriers that hinder the provision of physical health-care for people with SMI. Ultimately, this study adds to existing knowledge on the subject by applying the CA framework in the Israeli context, for the first time.

## Method

### Sample

This study was carried out in cooperation with Israel’s largest HMO, Clalit Health-services (CHS). After obtaining ethical approval, access to clinics was negotiated through regional managers. GPs were proposed by their clinic managers and were given a written description of the research. To ensure variability, clinics were recruited from a range of geographical regions and socio-economic profiles.

Patients, who were recruited through their GPs, were eligible to participate in the study if they had been diagnosed with schizophrenia or bipolar disorder, were forty years old or older, and could communicate in Hebrew. In addition, we limited participation to people diagnosed with a comorbid chronic physical illness (namely, Type-2 diabetes or a cardiovascular disease), to ensure that they required regular, periodic care from a GP. The final sample consisted of 10 GPs (2 men, 8 women), and 15 patients between the ages of 40 to 67 years (mean 49 years). Nine of the patients had been diagnosed with schizophrenia (5 men, 4 women), and six with bipolar disorder (4 men, 2 women).

### Data collection

We conducted semi-structured in-depth interviews with both the GPs and the patients. GPs were interviewed at their clinics, while the interviews with the patients took place at their homes, at coffee shops, or at their clinics, according to their preferences. Two different interview guides were used, one for each party. To ensure that issues elicited by the interview process were not overlooked, these guides were used flexibly, and emergent issues were allowed to guide subsequent dialogue.

The patient interviews began with an open-ended question – “Can you tell me the story of your physical illness?” – which allowed for the generation of salient experiences and memories related to both illness and treatment. This was followed by more specific questions: “Can you tell me about the last time you visited your GP?” “In your experience, how does your GP treat you?” “Can you tell me about the tests your GP asked you to do?” and “Do you have a message for physicians with reference to the treatment of patients with schizophrenia/bipolar disorder?”. When answers included content relevant to the research question, the interviewer asked additional questions to elaborate and clarify the patient’s views.

Interviews with GPs began with a general question – “In your opinion, in what way, if at all, do severe mental illnesses influence the treatment of physical illness?”, followed by more specific questions: “Can you tell me about a patient of yours who was diagnosed with schizophrenia or bipolar disorder?” “Can you tell me about your interaction with that patient?” and “How did your patient approach referrals you made for him?”. In addition, the GP was asked to describe other specific patients, a technique that facilitated the interviewer’s understanding of the varied experiences of the GP.

The interviews ended with questions about obstacles impeding the treatment of patients with SMI, and suggestions for needed improvements. All the interviews were audiotaped and transcribed verbatim. After each interview, the interviewers (MLA, RP, TR) met to reflect on the dialogue and evaluate the interview guide.

### Ethics, consent and permissions

The research was approved by the ethics committee of Clalit Health Services (CHS). We took extensive steps to protect the participants’ rights: an informed consent form was obtained all recordings were stored in a locked drawer, with access limited to the chief researcher (MLA); and to protect the patients’ confidentiality, pseudonyms were used in published materials.

### Data analysis

This research applied a systematic content and thematic analysis [22]. Themes were considered as labels that captured the essence of the discussion. The first step in the coding process was to create a coding frame. A preliminary coding frame was drawn up during an explorative, “open” stage, in which the researchers read the interviews carefully and searched for codes in the interviews themselves. This reflective process, which involved staying close to the data and to the theoretical aims of the research simultaneously [22], led to the identification of numerous codes that were integrated into conceptual categories via linking and splicing [22]. *Linking* refers to grouping codes to provide high-order themes that would allow for further abstraction and interpretation, yet simultaneously preserving the finer coding. *Splicing* refers to the use of clustered codes to create more general themes. Following this process, a final coding frame was drawn up and applied to all the interviews.

## Results

We identified multiple issues affecting desired health and personal outcomes at the patient, relational-societal and organizational levels. At the patient level, these issues included difficulties experienced due to psychiatric impairment, and low levels of functioning when trying to access appropriate health-care. Key themes at the relational-societal level included the patient-GP relationship, and stigmas associated with mental illness as key themes. Finally, issues identified at the organizational level included structural level impediments.

### Patient level

Both parties emphasized the general requirement of an active role for the patients within the health-care system, in order to ensure that they can receive the appropriate health services. The active role includes the need to be aware of one’s health condition and needs; to seek timely medical health-care; to actively address related administrative procedures; to understand and follow the recommendations of one’s GP, and so on. Both parties indicated that the ability of patients with SMI to take on such multi-level tasks may be compromised due to the severity of their psychiatric symptoms, the occurrence of acute episodes, and their functioning levels. For example, one interviewee, diagnosed with bipolar disorder and married to a woman diagnosed with bipolar disorder, described his efforts to help his wife take a health test:

*She is at home a lot. It is hard to get her out of the house. Last week, I took her for a stomach ultrasound, and it was very difficult. I had to set an alarm for 9 am, to wake her up and wash her and dress her, and to go out in the heavy traffic. It was raining, I had to find parking and find the place in the building and wait in line and [get to] the ultrasound room, and it was difficult, it was very difficult*. (Mr. and Mrs. Hassan, both diagnosed with bipolar disorder)

Access to, and utilization of health services demanded significant effort on the part of the patients, especially when their psychiatric symptoms were active. If the illness was in its active phase, the degree to which the patient could advocate for his/her own needs was restricted. In addition, one’s state of mental health seemed to influence the nature of the responses received from GPs and other medical professionals. This can be seen in the following quote:

*When I was in a poor mental state it was very difficult to deal with the procedure to fight for each pill, and sometimes they [pharmacists] insult you. Today I am more resilient (…) when you are insecure, not focused and with cognitive difficulties, you want to say a sentence and you are not sure what do you want to say. And then they shut you up, they shout and go.* (Ms. Tavori, diagnosed with schizophrenia)

### Relational-social level

Both parties described the importance of long-term, trusting relationships, to ensure that the health-care provided matched the patients’ mental status, needs, and abilities. Patients also emphasized the importance of having confidence in their GP, especially during periods when they experienced health and mental health crises. For example:

*During my last [depressive] episode, I told her* [my GP] *‘I’m falling’, and she said to me ‘You’ve come to the right place’. She kept in touch with me daily.* (Ms. Cohen, diagnosed with schizophrenia)

In this example, the patient shared her experience of attending her appointment in a state of stress and turmoil, which she described with the words “I’m falling.” She felt that her GP recognized the intensity of her emotional crisis and responded to her immediate needs, “to make sure that I will not fall,” thus providing her with a sense of security. Though this example is particularly poignant, given the effort made by the GP remain in daily contact, other patients similarly emphasized the importance of feeling cared for by their GPs during periods of crisis.

A number of GPs noted that the doctor’s level of familiarity with the patient, as well as certain characteristics of the GP-patient relationship, are crucial in determining the extent to which the delivery of services to patients with SMI can be efficacious. For example:

*I think that you need to be familiar with where the person is coming from; his family, what helps him to take his medications and remain stable, what knocks him off balance and… even to be a bit like a kindergarten teacher, to tell them, to ask them, to remind them, not to expect them to do it all alone. But also on the other hand, to give them autonomy, [to] give them autonomy and allow them to make decisions for themselves, give them the dignity as a person like anyone else, not to treat just the mental illness but see the person as a whole.* (Dr. Gurevitch)

Thus, both parties stressed the importance of developing a caring relationship, with a high level of familiarity, such that the GP becomes intimately aware of the patient’s complex needs, resources and abilities. The value of such relationships is twofold. First, it allows patients to feel comfortable with the GP, sharing experiences and information that the GP must know, in order to help them. In addition, the doctor’s genuine sense of care towards the patient’s welfare can help patients feel accepted and understood, with regard to their condition and its potential repercussions. This further supports adherence to treatment. For example:

*She [the GP] is nice, she is warmhearted and she is ok. She said to me also “why were there periods that you didn’t come to take the treatment?” She said to me “if it is about money, I will give you the money, don’t worry, we have a cashbox that will help you if you don’t have the money to buy medications.”* (Mr. Bar, diagnosed with schizophrenia and PTSD)

This quote demonstrates that a GP operating in a holistic, person-centered role can be essential in identifying the additional barriers that prevent successful engagement with the patient. Other patients, without trusting relationships with their GPs, described multiple difficulties with negative implications for the quality of care received. For example, one patient described a distance in the patient-GP relationship, which she connected to the lack of attention to her medical needs:

*She [the GP] has never taken an interest and asked me, “how do you feel?” Maybe there is a need for one less pill. She just prescribes the medication, and I take [the prescription] to the pharmacy.* (Ms. Oron, diagnosed with schizophrenia)

Another problem mentioned by the patients was when the GP did not recognize the difficulties experienced by the patient:

*Sometimes, I arrive at the last minute because it is urgent and I didn’t notice the time and then she [his GP] complains, “Why don’t you come on time? It’s impossible! I have a life too.” I don’t know, she is a bit [interviewee stutters] tough and not sensitive (…) when I come I worry, I am worried that she will send me away because it is as though I have come to disturb her.* (Mr. Reshef, diagnosed with bipolar disorder)

From the perspectives of these patients, the two GPs discussed above were unable to recognize their patients’ needs and difficulties. The absence of trust on the part of the patient, therefore, reduces the chances that the patient will feel comfortable enough to provide a complete account of his/her health status. Notably, some of the patients did not attend subsequent appointments with their GPs, due to the uncomfortable nature of their relationships with their GPs. The patients believed that a main cause of such negative relationships was the stigma associated with SMI.

#### Stigma of SMI

GPs and patients discussed the prejudice manifested against people with SMI as a dominant norm, one negatively affecting patients’ motivation to utilize available health services. One manifestation of this prejudice was that of downplaying patients’ physical complaints, attributing their health conditions to an expression of their mental health status:

*She thinks that if I see a psychiatrist then everything is… even if I come to her [*i.e.*, the GP] then everything is a psychiatric [problem]*. (Mr. Amos, diagnosed with schizophrenia)

Some of the GPs addressed this issue candidly:

*I think that there is [a tendency] to put less effort in preventive medicine and also in the treatment of the psychiatric patient, to deal less with complaints and symptoms. It is easier to think that these complaints are not genuine – not genuinely physical – even though they are. That’s my general feeling*. (Dr. Albez)

Thus, the at-times stigmatic notions of SMI emerge, affecting the treatment options and explanations offered to individuals with SMI by their GP, as well as how patients’ complaints are perceived by GPs.

Another form of impaired health-care provision was reflected in the limited provision of services and referrals. This tendency was driven by the widely held notion that patients with SMI find it difficult to take up and utilize available health services. To help them avoid the hardships and difficulties associated with approaching and accessing health services, physicians tended to refrain from suggesting certain medical procedures to their patients. For example:

*Does it influence us that they are [mentally] sick? Does it affect our decision-making regarding their treatment? Sometimes maybe yes, because often these people are quite lonely and when you know that they…either will not be able to even book an appointment or will encounter difficulties and will not find the right address, or that they don’t have someone to accompany them and they will miss their appointment, then sometimes you even consider making as few referrals and examinations as possible.* (Dr. Braun)

Here, Dr. Braun describes a vicious cycle, one in which physicians’ awareness of the difficulties experienced by SMI patients in utilizing available health services leads to GPs suggesting fewer services to those patients. Although many physicians may believe that they are acting in the best interests of their patients, their decisions deprive their patients of the opportunity to approach the services in the first place.

### Organizational level

In addition to the above, two kinds of structural-level impediments were described by the GPs: the absence of continuous patient monitoring, and the lack of resources specifically dedicated for psycho-social interventions.

#### Monitoring

Lack of SMI patient health monitoring was referenced by the GPs as a significant shortcoming in current mental health-care protocols. Where implemented for physical illnesses (e.g., diabetes), patient health monitoring enables continuous and comprehensive health-care, while at the same time helping to verify that the health-care provision in question is both appropriate and timely.

*It is even possible that you have in your list of patients, people with schizophrenia that you have never seen them… that… they do treatment in a psychiatric clinic or in the ministry of health and you never see them … then it is possible that chronic illness are hidden or maybe not… but you don’t even know of their* [patients’] *existence*. (Dr. Brahman)

In common with other GPs, here Dr. Brahman describes patients who are registered but never visit the clinic. GPs also discussed how the lack of monitoring affected patient compliance and adherence, with regard to compliance with prescribed medications, and ongoing psychiatric and general health-care management.

Some GPs observed how the protocols for other health conditions included the creation and maintenance of electronic databases. Electronic databases provide periodic updates for physicians about their patients’ health-care, facilitating monitoring functions. Such electronic databases do not exist for the population of SMI patients.

*There are, let’s say, diabetes reports, blood pressure reports, I can… see who are my diabetes patients and who did not do the test, this is very important to the management (…) But I don’t have a report of schizophrenic patients, so…but… there are indirect ways, if I have a patient who is both schizophrenic and diabetic and he didn’t do the test so… [I can say] “Ah! I really haven’t seen him for long time, let’s get his file and see what has happened.”* (Dr. Gabay)

Drs. Gabay, and other GPs, imply that health system policy must first be changed so that people with SMI can be identified as part of a high-priority population, and so that their utilization of health-care services can be monitored.

#### Resources for psycho-social intervention

GPs acknowledged that they do not always know how to help their patients, as they do not understand the difficulties specific to their SMI, or how to overcome those difficulties. This scenario, in which the GPs feel that they lack the competence to help their patients, is a source of frustration for the doctors. Moreover, the GPs are wary not to subject their patients to unnecessary stress, are unsure of how to support patients’ life style changes, and are frustrated by what manifests as the patients’ lack of motivation. Most of all, however, GPs feel helpless when confronted with patients who regularly miss their appointments. Some GPs discussed the possibility of receiving supplementary assistance from professionals from other fields, in order to improve the treatment they can offer their patients:

*In the past we thought that it will be good if we can have a psychiatrist in the clinic (…) I need to know how to treat him [a patient with SMI], and how to cope with him and so on. How to solve the mental together with the physical difficulties, how to cooperate with him better, this is the aim. (Dr. Braun).*

*A lot of responsibility and not enough tools, the main tool that is needed, of course, is time and second [tool is] availability, and direct contact with a psychiatric consultant.* (Dr. Frith)

Drs. Braun and Dr. Frith both feel strongly that interdisciplinary consultation, in the form of psycho-social intervention, could help improve the levels of support they are able to offer their patients, and that the support of such professional intervention should be an integral responsibility of the health system. Currently, there is no systematic mechanism for supporting GPs’ attempts to improve the treatment they can provide to patients with SMI.

GPs and SMI patients alike note that more time needs to be devoted to doctor-patient interaction, and the importance of coordinating the treatment given the patient by the different professionals responsible for him/her –psychiatrists, social workers and family members, amongst others. But the establishment of collaborative partnerships with other professionals or with the patient’s family members demands time and effort. Physicians often cannot afford the time necessary to manage such complex interventions.

## Conclusion

While inequality in the provision of physical health-care for people with SMI is well documented, in-depth knowledge of the personal experiences of these barriers is limited. The current study identified health capability sets suggesting a conversion-handicap [19], especially with regard to disadvantages in accessibility to physical health-care facilities. These included complex encounters and underutilization of physical health services. We contextualize and interpret these using CA concepts of conversion factors.

### Personal conversion factor

Psychiatric symptoms and impaired functioning were evident as personal conversion factors. Interviewees reported varying levels of access to physical health-care, according to the phases of their illness (e.g., acute phase, remission). Symptom severity negatively affected the patients’ ability to keep to meetings and respond constructively to incidents of discrimination and stigma, which had a negative effect on their health and mental health states. Such impediments limited the extent to which patients could take up their GP appointments and regular checkups [17, 23]. Thus, in the framework of CA, severe mental illness-related impairments constituted negative personal conversion factors, that diminished the patients’ ability to translate existing health resources and services into desirable health outcomes.

### Social conversion factor

The next level of conversion extends beyond accessibility to the social conversion factor, which resonated in the findings via the types of relationships formed between patients with SMI and their GPs. An absence of personalized relations and/or the GPs’ belief that the they lacked the resources necessary to help the SMI patient hindered the effective use of their services and knowledge. Other studies have identified the centrality of the GP-patient relationship, with regard to improved health and rehabilitation outcomes in the SMI population [17, 24–26].

Our study also showed the role of stigma in intensifying negative patient-GP relationships. GPs also minimized contact with patients based on negative attitudes and beliefs. Other reports indicated the existence of stigmas associated with mental illness in Israel [27], and to the negative effects that stigma and discrimination could have on the patients’ relationships with their GPs [28], psychiatrists [29], and other health practitioners [7, 25]). Negative attitudes toward people with SMI led to poor GP-patient communication, and the provision of less-than-adequate care and poor-quality services [17, 28].

### Environmental conversion factor

Finally, the study findings contour multiple environmental conversion factors lessening the effectiveness of physical health-care service delivery on behalf of GPs. These factors included the absence of proper health monitoring, lack of psychiatric knowledge on the part of GPs, and time constraints preventing GPs from providing comprehensive care to SMI patients. Structural-level shortcomings related to power relations have been reported previously within SMI population in other domains [30]. Here too, in relation to health-care, we suggest that the institutional structures and norms override conversion-handicap [31]. Due to the social power relations (also emphasized in CA), we point to the implicit beliefs of GPs and lacunas in health monitoring systems that have negative, albeit inadvertent, consequences on the capability sets of people with SMI.

### Limitations

This study focused on the capability of “bodily health,” which refers to the ability to have good health [19]. The focus on a single aspect of health functioning (bodily health) might neglect Sen’s holistic view of a person’s health as contextualized within broader scopes, including psychological wellbeing and quality of life. A fuller assessment of the functioning and well-being of people with SMI can address issues related to access and utilization of leisure activities and social networks. In this sense, future research can extend the focus, to include the interface of health services with rehabilitation services. This could help attain a more complete understanding of the achievement of functioning outcomes, involving the attainment of a well-rounded, positive life for persons with SMI. An additional limitation of the current study is the lack of representation of minority groups with SMI. All the participants were Hebrew speakers. Future studies may sample other groups suffering from SMI in Israel, such Arab-Israelis, first- and second-generation migrants of Ethiopian origin, and the ultra-Orthodox community, to maximize the represented variability. These subgroups may have overlapping as well as distinct experiences with GP services, specifically with regard to socio-cultural factors, that may play a critical role in their influence on conversion factors. Indeed, a recent study reported on the double-disparities of health services for Arab-Israelis with schizophrenia [32].

In addition, the inclusion of both patients and physicians relied on the cooperation and knowledge of local key informants. This might imply that the GPs whom we interviewed were those with a greater interest in the quality of care afforded their patients, or who were more aware of the topic. In addition, the possibility that the patients we interviewed were those who had better relationships with their GPs cannot be ruled out. Furthermore, it is possible that the patients interviewed might have tried to be more positive about their GPs, given the apprehension that their GPs might, with time, get to know what they have said. Combining these factors raises the possibility that we might have been exposed to disproportionally positive experiences of GP-patient interactions. However, as in our interviews, we have heard both positive and negative experiences, and we assume that the conceptual model we have constructed is not biased.

## Discussion

In 2015, a reform of provisions for mental health-care was launched in Israel with the goal of unifying community health-care and mental health services. Consequently, more individuals with SMI are expected to turn to community health services for their health needs. In light of our findings, GPs will need much support and guidance in order to ensure adequate care for patients with SMI. One way of addressing these challenges would involve developing a distinctive role within the HMOs, to communicate knowledge about psychiatric care and rehabilitation services to GPs. A study of the evolution of the role of GPs following the reform has shown that to feel comfortable about asking for help and advice about mental health provisions for patients with SMI, GPs need a personal connection with a specific mental health professional [33]. Such a role can help to address the environmental conversion factors – namely providing access to knowledge, and better coordination of the special issues related to health services for individuals with SMI. In Israel there are a substantial number of professionals (social workers, occupational therapists, psychologists etc.) with significant experience in the field of psychiatric rehabilitation, informed about the enduring problems of SMI patients that impede their health-care, and aware of eligibility criteria for community and hospital services.

In other countries, some programs were found helpful for community services. For example, in Massachusetts a timely telephonic psychiatric and clinical guidance service was made available to primary care providers (PCPs) treating children with mental health problems. These initial phone consultations enabled PCPs to provide in-person psychiatric or clinical assessment, transitional therapy, and/or facilitate linkage to community resources [34].

In addition, based on our findings and in order to reduce the health-care inequalities experienced by people with SMI in Israel, we propose that multiple aspects related to the different conversion factors are addressed: **1**. GPs should take a proactive approach in monitoring patients’ health status and utilization of services. **2**. Policy makers must officially acknowledge that people with SMI constitute a vulnerable population at risk, and allocate increased time for GP-SMI patients consultations. **3**. GPs should receive training and support for enhancing their communication skills with SMI patients. **4**. Better collaboration and coordination between GPs and other mental-health professionals is necessary. (To achieve this goal, the financial and structural barriers to general and mental-health collaboration should be identified and overcome); and finally, **5**. The dominant norm of prejudice against people with SMI must be reduced through educational interventions with health professionals. One way to achieve this is through GP training and exposure to individuals with SMI who are further along in their recovery. This can be effective in creating more positive relationships and optimistic outlook of the GPs. Personal contact is known to be effective in reducing stigma [34]. Notably, a workforce of mental health peer supporters within the psychiatric services is in the process of being developed; given the ongoing mental health reforms in Israel, it will be practical to direct training and resources to GPs as well [33].
